# Different Transcriptional Profiles of RAW264.7 Infected with *Mycobacterium tuberculosis* H37Rv and *BCG* Identified via Deep Sequencing

**DOI:** 10.1371/journal.pone.0051988

**Published:** 2012-12-19

**Authors:** Fengguang Pan, Yaya Zhao, Seng Zhu, Changjiang Sun, Liancheng Lei, Xin Feng, Wen yu Han

**Affiliations:** 1 Laboratory of Food Safety and Hygienic Inspection, Jilin University, Changchun, Jilin, People’s Republic of China; 2 College of Animal Science and Veterinary Medicine, Jilin University, Changchun, Jilin, People’s Republic of China; Queen Mary University of London, United Kingdom

## Abstract

**Background:**

The *Mycobacterium tuberculosis* H37Rv and BCG effects on the host cell transcriptional profile consider a main research point. In the present study the transcriptome profiling analysis of RAW264.7 either infected with *Mycobacterium tuberculosis* H37Rv or BCG have been reported using Solexa/Illumina digital gene expression (DGE).

**Results:**

The DGE analysis showed 1,917 different expressed genes between the BCG and H37Rv group. In addition, approximately 5% of the transcripts appeared to be predicted genes that have never been described before. KEGG Orthology (KO) annotations showed more than 71% of these transcripts are possibly involved in approximately 210 known metabolic or signaling pathways. The gene of the 28 pathways about pathogen recognition receptors and *Mycobacterium tuberculosis* interaction with macrophages were analyzed using the CLUSTER 3.0 available, the Tree View tool and Gene Orthology (GO). Some genes were randomly selected to confirm their altered expression levels by quantitative real-time PCR (qRT-PCR).

**Conclusion:**

The present study used DGE from pathogen recognition receptors and *Mycobacterium tuberculosis* interaction with macrophages to understand the interplay between *Mycobacterium tuberculosis* and RAW264.7. Meanwhile find some important host protein which was affected by *Mycobacterium tuberculosis* to provide evidence for the further improvement of the present efficacy of existing *Mycobacterium tuberculosis* therapy and vaccine.

## Introduction

Tuberculosis (TB) is one of the most common and widely distributed bacterial diseases where one third of the world population infected and causing 1.7 million deaths annually [1], [2]. The causative agent of TB is *Mycobacterium tuberculosis* which can survive in host under a dormant state to delay its multiplication under adverse metabolic conditions for years [3]. TB continue to increase annually in spite of efficient chemotherapies which have been developed [4]. More seriously, strains of *Mycobacterium tuberculosis* which is resistant to a range of antimicrobials have emerged [5].

To face this problem, much research effort has been made in recent years towards understanding *Mycobacterium tuberculosis* and especially the interplay between M*ycobacterium tuberculosis* and host cells, expecting it will lead to the development of novel therapies and vaccine. DNA microarrays has been used to study the transcriptional profiling of *Mycobacterium tuberculosis* to investigate how *Mycobacterium tuberculosis* can destroy the host immune system response by Ward SK, 2010 [6]. However, the opportune response of host to *Mycobacterium tuberculosis* and the antigens they confront are still unclear. Simultaneously, the innate immune response to pathogenic bacteria and the subsequent adaptive immune response to eliminate the pathogens which can protect the host from pathogenic bacterial invasion have not been expound systematically [7].

As we known, H37Rv is a virulent *Mycobacterium tuberculosis* laboratory strain, where BCG is a widely used vaccine against *Mycobacterium tuberculosis* [8]. Thoroughly understanding the bacterial factors that affect some important pathways such as phagosomal antigen processing may help in improving strategies to eliminate *Mycobacterium tuberculosis*. H37Rv and BCG may have different effects on genes under adverse metabolic conditions positively selected in *Mycobacterium tuberculosis*. The functional discussion of these findings may imply that these selected genes may be involved in antigen variations and immune evasions of *Mycobacterium tuberculosis*. However, limited studies have reported on the large-scale the identification of immune-related genes at the genome or transcriptome levels in RAW264.7, which become a general cell model for TB studies nowadays, because current high-throughput deep sequencing technologies are insufficient [9], [10]. The DGE system is a tag-based transcriptome sequencing approach in the production of technical innovation of RNA deep sequencing technologies; this system removes the limitation of study about immune-related genes and functional transcription complexes [11], [12]. During the DGE approach, clean tags strained through raw tags with certain end nuclease should be mapped to the reference database. The degrees of expression of the actual total genes in the sample are the criterion defined by the quantity of each mRNA molecule yield from each gene in this approach [13]. Due to reasonable agreement on cell biology, DGE is mainly used to study the comparative gene expression [10], [14]. The DGE approach can also be applied in the field of cellular, disease, and immune defense.

The present study is the first to conduct a transcriptome profiling to characterize the relationship between RAW264.7 gene expression profiles after *Mycobacterium tuberculosis* H37Rv and BCG infection using the DGE system. Furthermore, a round survey of the anti-bacterial immune defense gene activities in RAW264.7 can contribute to the in-depth investigation of candidate genes in the host immunity defense against *Mycobacterium tuberculosis*. The results of the present study may help in improving the current understanding on host–pathogen interactions.

**Figure 1:**
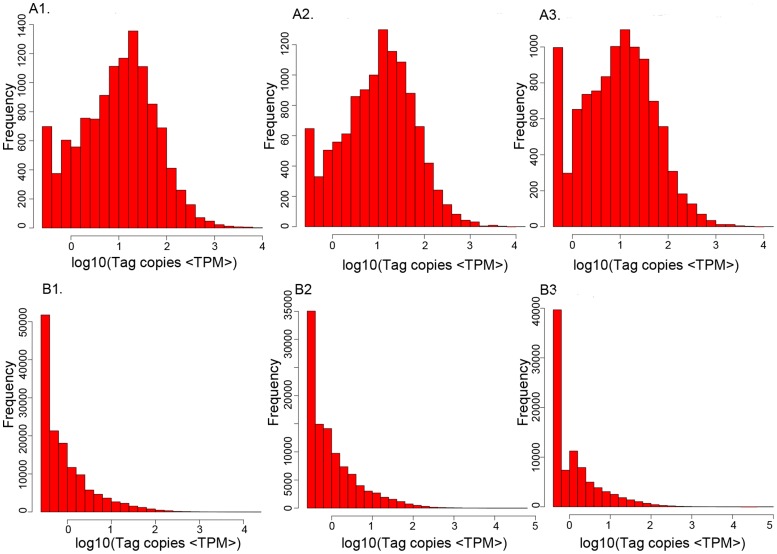
Distribution of gene expression (A) and tags (B) between experimental and control groups. Panel A represented the distribution of gene expression in the H37Rv-treated library (A1), BCG-treated library (A2), and control library (A3). Panel B represented the distribution of tags in the H37Rv-treated library (B1), BCG-treated library (B2), and uninfected library (B3). TPM represented tags per million. Results showed that the distribution of tags matched the distribution of genes for each group. Furthermore, the frequencies of tags or genes decreased with increasing number of tags or gene expression. Results suggested that these DGE dates are significant.

## Materials and Methods

### Cell and Bacterial Cultures

RAW264.7 (ATCC TIB-71) was purchased from the National Institute for the Control of Pharmaceutical and Biological Products of china. RAW264.7 cells were maintained in supplemented Dulbecco’s modified eagle medium (DMEM) composed of 10% fetal bovine serum, 0.2 mM L-glutamine, 100 µg streptomycin/mL, and 100 U penicillin/mL (Sigma, St. Louis, Mo.) at 37°C with 5% CO_2_ [15].

**Table 1:** Major characteristic of DGE libraries and tag mapping to the UniGene transcript database.

	*the BCG-infected library*				*the H37Rv-infected library*			
	*Distinct Tag*	*%*	*Total Tag*	*%*	*Distinct Tag*	*%*	*Total Tag*	*%*
Raw data	306688	100	5759300	100	303581	100	5773517	100
Low Quality Tag	0		0		0		0	
Tags containing N	774	0.25	940	0.02	893	0.29	1053	0.02
Adaptor tag	82	0.03	94	0	84	0.03	89	0
Tag copynum <2	202170	65.92	202170	3.51	166118	54.72	166118	2.88
Clean tag	103662	33.8	5556096	96.47	136486	44.96	5606257	97.1
Copynum >2	103662	100	5556096	100	136486	100	5606257	100
Copynum >5	13385	12.91	101008	1.82	15986	11.71	120238	2.14
Copynum >10	8809	8.5	129049	2.32	10198	7.47	148787	2.65
Copynum >20	7384	7.12	236865	4.26	8561	6.27	274419	4.89
Copynum >50	3872	3.74	273595	4.92	4154	3.04	295194	5.27
Copynum >100	6106	5.89	4638534	83.49	6432	4.71	4520966	80.64
Tag mapping								
All mapping	67106	64.74	4723249	85.01	77287	56.63	4731204	84.39
Unambiguous mapping	60472	58.34	3197086	57.54	70300	51.51	3376934	60.24
Unknown tag	14728	14.21	477629	8.60	23008	16.86	523621	9.34

All Mapping represents the number of all tags mapped to the UniGene virtual tag database, clear-cut Mapping represents the number of clear-cut tags mapped to the UniGene virtual tag database, clear-cut tags indicate the tags matched only to one gene. Examination confirmed that the quality of the data were up to specification suggesting that DGE data are significant.

### Bacterial Cultures and Bacterial Growth Assay in vitro

H37Rv (ATCC27294) and BCG (ATCC19274-50) were purchased from the National Institute for the Control of Pharmaceutical and Biological Products of china. The H37Rv and BCG were grown as previously described using standard methods [16]. Bacterial growth assay in RAW264.7 was done as previously described [17].

**Figure 2:**
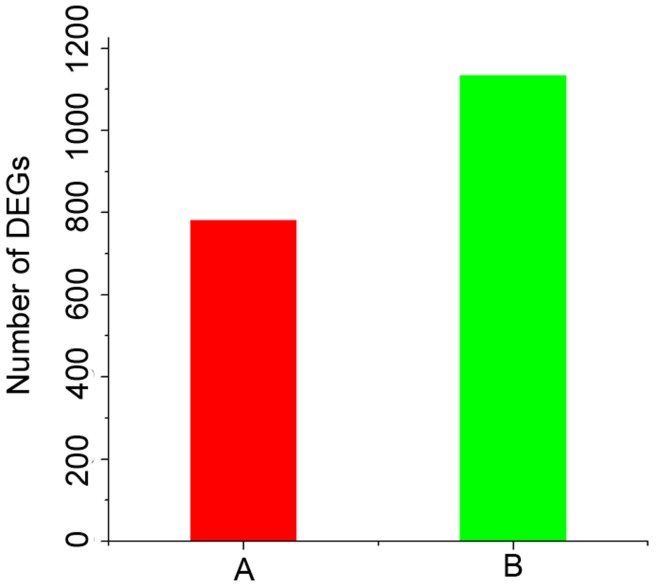
DEGs of H37Rv-treated and BCG-treated libraries. The red and green bars represented the upregulated and downregulated DEGs in the H37Rv-treated library condition relative to the BCG-treated library, respectively. Results showed that the number of significantly DEGs between two samples was 1,917, where 1,135 genes and 782 genes were up-regulated and down-regulated, respectively.

### *Mycobacterium tuberculosis* Infection

Bacteria were grown to mid-log phase before being pelleted and resuspended in RPMI 1640 medium plus 10% fetal bovine serum and HEPES. RAW264.7 cells were plated in 75 cm flasks in supplemented DMEM without fetal bovine serum and antibiotics 1 day prior to infection at a concentration of 1×10^6^ per flask. Macrophages (6×10^6^ cells per flask) were infected with different bacterial strains at a multiplicity of infection (MOI) of 5 for 4 h [18]. Following this, colony counts were done as previously described [18].

**Figure 3:**
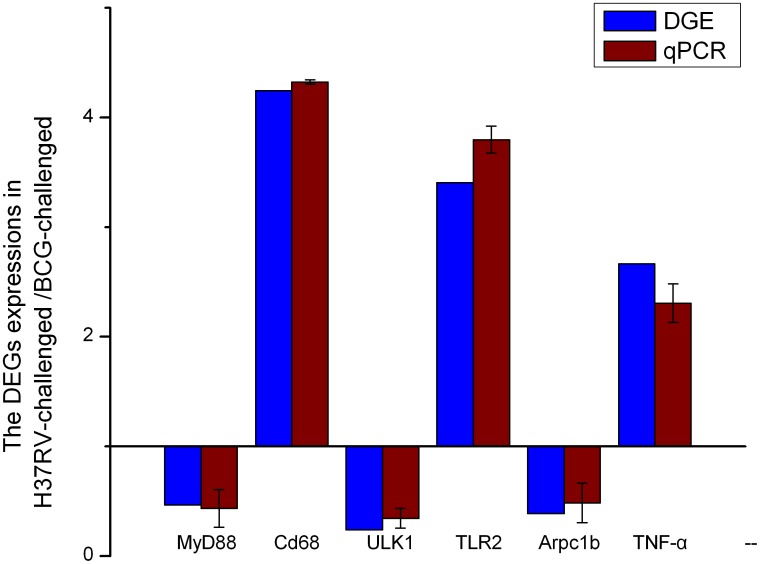
Validations of DGE data via qPCR. The vertical axis indicated the fold change of the expressions of DEGs in the H37Rv-treated library compared with those in BCG-treated. DGE indicated the RNA samples from the pooling samples that were used. qPCR indicated the RNA samples from independent RNA extractions from biological replicates. The error bars represented SE. Pearson’s correlation coefficient (r) showed that both the DGE and qPCR data were highly correlated, where the DEGs had a high consistency and the r values ranging from 0.681 (TLR2) to 0.995 (MyD88) between the two methods suggesting that the DGE results are significant.

### RNA Extraction

The total RNA was extracted from RAW 264.7 cells infected with *Mycobacterium tuberculosis* H37Rv and BCG using standard protocols (Trizol) [19]. To eliminate the potential genomic DNA contamination, RNase-free DNase I (Qiagen) was used according to the manufacturer’s protocol. The RNA concentrations were evaluated using Agilent 2100 Bioanalyzer (Agilent Technologies). A 1.5% (w/v) agarose gel was used to analyze the integrity of RNA.

**Figure 4:**
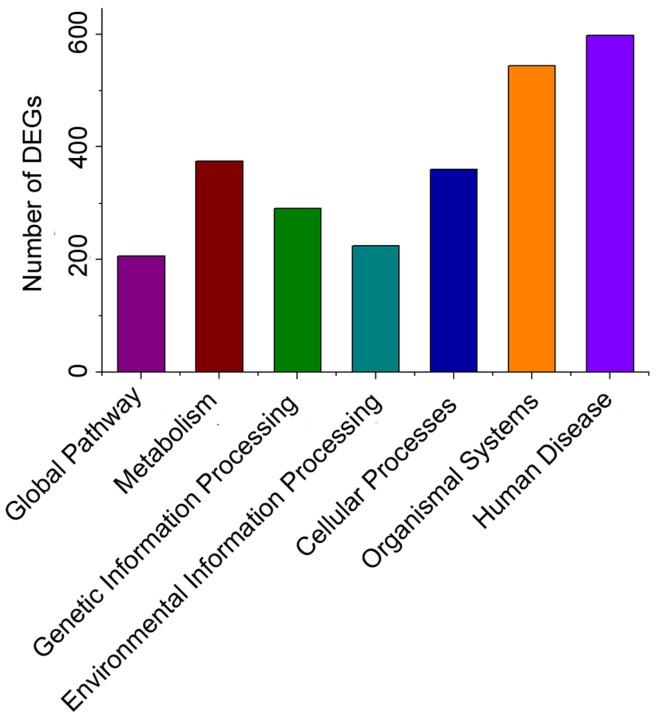
Categories of DEGs based on KEGG. The pathways distribution showed in Figure 4: among the 1,917 DEGs, 1,371 can be mapped into 210 pathways which be classified functionally into seven categories: global pathways, metabolism pathways, genetic information processing pathways, environmental information processing pathways, cellular processes pathways, organismal systems pathways, human diseases pathways based on the KEGG function classification, where the human diseases pathways showed highest amount of genes measured as 603 genes, while Organismal systems pathways were 562, Genetic information processing pathways were 385, metabolism pathways were 383, cellular processes were 361, environmental information processing pathways were 224 and Global pathways were 212. Results suggested that after H37Rv or BCG infecting macrophages or possibly DCs, the global pathway, metabolism, genetic information processing, environmental information processing, cellular processes, and organismal systems all adopt some changes as shown above.

### Library Construction and Sequencing

Illumina Gene Expression Sample Prep Kit and Solexa Sequencing Chip (flowcell) were used for the library construction and sequencing [20]. The main instruments used were the Illumina Cluster Station and Illumina HiSeq™ 2000 System. Library construction and sequencing were done as previously described [20].

**Figure 5:**
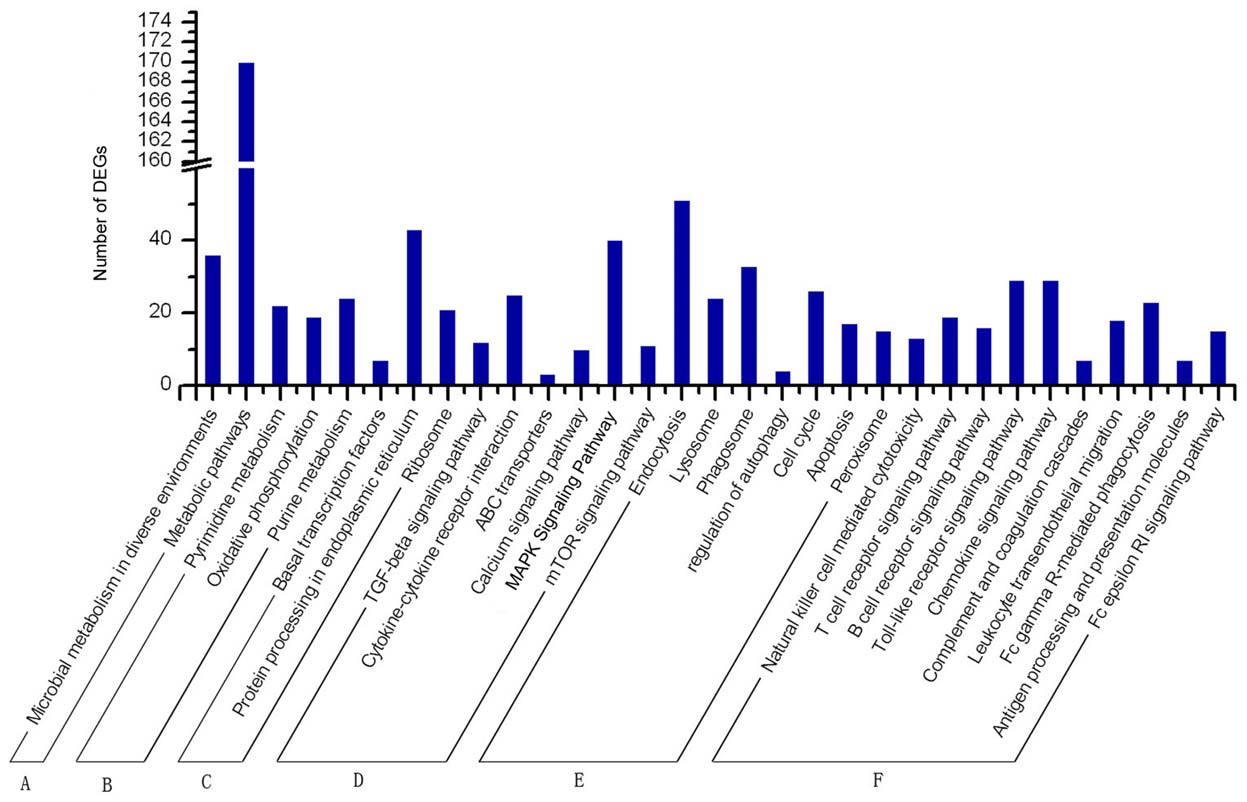
Categories of some DEGs based on KEGG. A) Global pathways; B) Metabolism pathways; C) Genetic information processing pathways; D) Environmental information processing pathways; E) Cellular processes pathways; F) Organismal Systems pathways. We from our point of view selected 28 pathways based on the potential role and interaction with *Mycobacterium tuberculosis*, where we tried to make the selection cover all the stages of infection and immune response, then made further study on the selected pathways, where we grouped it into six categories based on KEGG, and illustrate its distribution as shown in Figure 5. Results suggested that in these 28 selected pathways the DGEs mainly distributed in global pathways, cellular processes pathways and organismal Systems pathways.

### Analysis and Mapping of DGE Tags

Firstly, dirty tags were filtered as previously described [21], to acquire clean tags containing CATG and 21 bp tag sequences for mapping the DGE tags. Subsequently, the clean tags were mapped to the *Mus musculus* reference database, allowing one nucleotide mismatch at most, for the study of tag annotation. The clean tags were labeled as clear-cut clean tags. The saturation analysis of each sequence was performed as described previously [20] to verify whether the number of detected genes continue to increase with increasing sequencing amount (total tag number). For the gene expression analysis, the number of clear-cut clean tags for each gene was calculated and normalized to tags per million (TPM).

**Figure 6:**
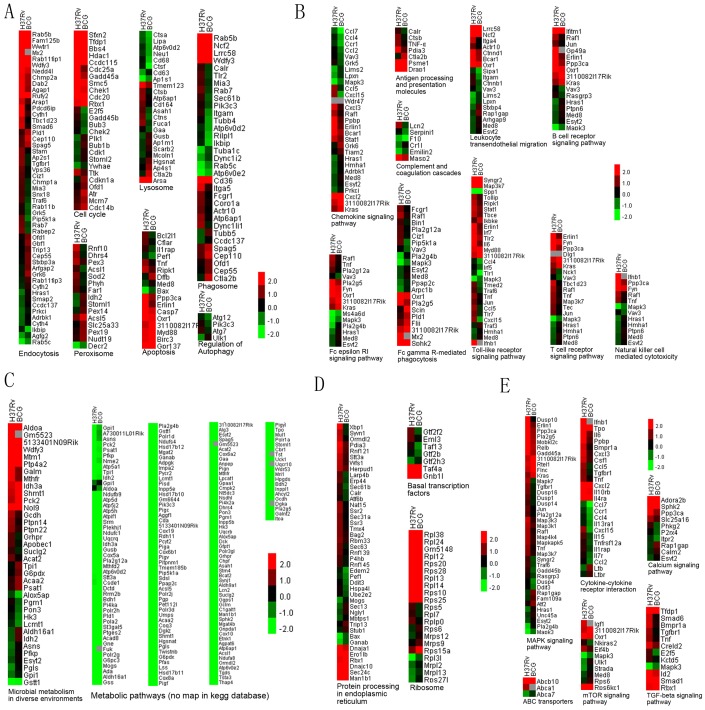
Hierarchical clustering and Tree View analysis. Figure 6A. The metabolism pathways analysis. Figure 6B. The genetic information processing pathways analysis data. Figure 6C. The Environmental information processing analysis. Figure 6D. The cellular processes analysis Figure 6E. The Organismal systems analysis. 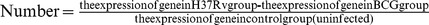 The up regulated genes showed value over zero, while the down regulated genes showed value range from 0 to −2. Results showed that the most up regulated genes belongs to the following pathways: cellular process pathways, such as cell cycle and apoptosis; environmental information processing pathways, such as MAPK signaling pathway, TGF-beta signaling pathway; genetic information processing pathways such as ribosom; global pathways such as microbial metabolism in diverse environment; organismal systems pathways, such as T cell receptor signaling pathway, antigen processing and presentation molecules, while the most down regulated genes belongs to the following pathways: cellular process pathways, such as phagosome, endocytosis and autophagy; environmental information processing pathways, such as calcium signaling pathway; global pathway such as metabolic pathways; organismal systems pathways, such as Fc epsilon RI signaling pathway (as shown in Figure 6, Table S3, and Table S5). Results suggested that if we want to improve present tuberculosis therapy, we may pay more attention to the function of these DGEs to effectively control *Mycobacterium tuberculosis* infection.

### Evaluation of the DGE Libraries

To compare the gene expression differences, the tag frequency in each DGE library was statistically analyzed according to the method described by Audic and Claverie [22]. To determine which genes are significantly differentially expressed in the libraries, a *P*-value of <0.005, a false discovery rate (FDR) of <0.01, and an estimated absolute log2-fold change of >0.5 were selected as threshold.

**Figure 7:**
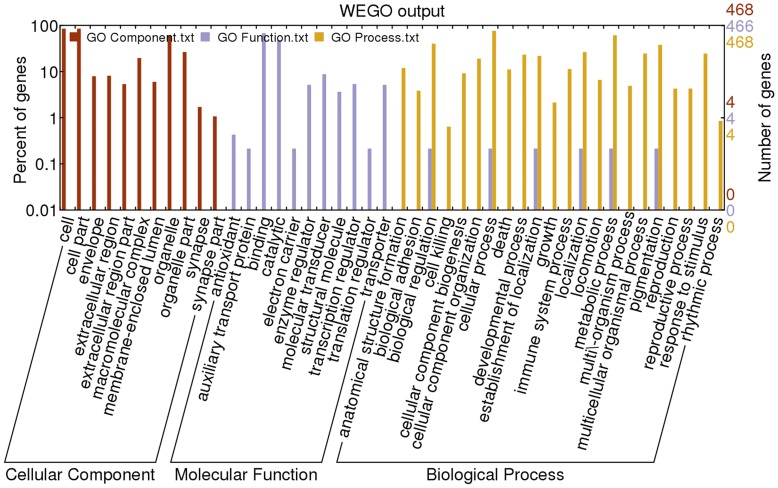
GO categories of the part unigenes. Red represented GO Component, blue represented GO Function; yellow represented GO Process. The DEGs from the selected 28 pathways were analyzed by the WEGO analysis. Results showed that about 468, 466, and 468 genes could be annotated in GO component, GO function, and GO process based on sequence homologies, respectively. In the three main categories (cellular component, molecular function, and biological process) of the GO classification, “cell,” “cell part,” “binding,” “catalytic,” “metabolic process,” and “cellular process” were dominant.

### Quantitative Polymerase Chain Reaction (qPCR) Validation

Total RNA was extracted as described for the DGE library preparation and sequencing [19]. MyD88 [23], Cd68 [24], ULK1 [25], TLR2 [26], Arpc1b [27]and TNF-α [26]were randomly selected to validate the DGE results via quantitative reverse transcriptase-PCR. The sequences of the specific primer sets are given in Table S1. Glyceraldehyde-3-phosphate dehydrogenase (GAPDH) was used as an internal gene [27]. qPCR was performed using the SYBR Premix Ex Taq Kit (TaKaRa) according to the manufacturer’s protocol.

**Table 2:** *Mycobacterium tuberculosis*-influenced immune-relevant genes in RAW264.7.

*Symbol*	*TPM-* *H37Rv*	*TPM-BCG*	*Symbol*	*TPM-* *H37Rv*	*TPM-* *BCG*
Recognition and ingestion			Rab5		
Toll-like receptor signaling pathway			Rab5b	27.11	5.22
Tlr1	6.78	1.08	Rab5c	50.48	121.85
Tlr2	197.28	57.95	Rab7		
Tlr7	22.3	5.76	Rab7	26.04	6.3
Tbce/Tlr5	22.83	4.5	Rabep2	10.17	1.98
Tollip	88.12	38.7	Lysosome		
Myd88	14.27	30.6	Atp6ap1	98.1	34.56
Hmha1	21.94	51.12	Atp6v0d2	133.96	48.96
Map3k7	60.29	27.72	Ap1m1	45.48	124.55
Mapk3	12.13	35.28	Ap1s1	37.64	102.05
Ccl4	3270.27	1060.1	Ap4s1	15.34	48.6
Ccl5	22.83	4.5	Lysosomal membrane protein		
Irf5	55.83	23.94	Cd68	5.35	1.26
Irf7	45.31	15.48	Scarb2	27.11	62.99
Stat1	58.51	28.8	Cd63	163.39	505.57
Tnfα	299.13	112.13	Mcoln1	16.23	33.3
Traf3	8.56	19.62	Lysosomal acid hydrolase		
Traf6	21.58	6.66	Protease		
Il6	6.06	0.9	Cathepsins		
Ifnb1	3.03	0.01	Ctsf	12.31	1.98
Ikbke	45.48	124.73	Ctsb	2195.4	622.2
Complement receptor			Ctsa	300.74	133.01
Cr1l	99.71	171	Ctla2b	0.54	3.6
cytokine and cytokine receptor			Glycosidases		
Il6	6.06	0.9	Gaa	37.46	16.74
Il15	12.84	3.78	Neu1	38.71	15.84
Tnf	299.13	112.13	Gusb	200.31	604.38
Tnfaip8	16.23	41.58	Sulfatase		
Tnfaip6	2.5	0.01	Arsa	4.64	14.58
Tnfaip3	60.82	8.46	LIPASE		
Ifnb1	3.03	0.01	Lipa	87.22	41.22
Tgfbr1	17.3	4.5	Ceramidase		
Tnfrsf12a	10.7	3.78	Asah1	122.18	32.94
Traf6	21.58	6.66	Fc gamma R-mediated phagocytosis		
Ripk1	71.35	28.26	Cfl1	852.08	1741.33
Traf7	43.88	18.54	Arpc4	32.29	84.41
Traf3	8.56	19.62	Scin	4.82	32.04
Endosome			Med8	49.59	114.83
EEA1			Hmha1	21.94	51.12
Cep110	4.28	12.6			
Cep55	4.64	12.24			

TPM represented tags per million. H37Rv (QD) represented the group in which RAW264.7 were treated with H37Rv; BCG (RD) represented the group in which RAW264.7 were treated with BCG. TPM represented tags per million. Symbol, Gene symbol (Brief gene description can be found in Table S2). Results showed that, the expression of some DEGs which involved in recognition and ingestion, complement receptor, cytokine and cytokine receptor, Endosome, Lysosome, lysosomal membrane protein, Lysosomal acid hydrolase and Fc gamma R-mediated phagocytosis, suggesting that these genes may be the involved in the main host protein which was affected by H37Rv or BCG.

### Gene Ontology and Kyoto Encyclopedia of Genes and Genomes (KEGG) Functional Enrichment Analysis for Differentially Expressed Genes (DEG)

During gene ontology analysis, the gene sets with *P*<0.1 should be deemed significantly enriched. An FDR of <0.1 must be considered for the KEGG enrichment analysis [21] [28].

### Hierarchical Clustering and Tree View

The genes and their library expressions were analyzed using the CLUSTER 3.0 (http://bonsai.hgc.jp/~mdehoon/software/cluster/software.htm) available on the Internet and the Tree View tool (http://www.yiiframework.com/forum/index.php?/topic/9180-the-tree-view/).

## Results

### Analysis of DGE Libraries

The global gene expression profiles of RAW264.7 to *Mycobacterium tuberculosis* were measured using the Solexa/Illumina’s DGE system. Two separate RAW264.7 libraries were sequenced using massively parallel sequencing on the Illumina platform after infection with H37Rv or BCG. The summary of these two libraries is shown in Table 1 and Figure S1, where the total clean sequence tags numbers of the two libraries was 5.6 million (Figure 1) and 120074 of which were distinct clean tags sequences. The proportion of the amount of distinct tags to the total tags in H37Rv challenged was 5.26% and in BCG challenged libraries was 5.33%. In saturation analysis of the capacity of libraries, with more and more sequence tags, the amount of new distinct tags was decreasing, in the condition that the amount of total sequence tags was big enough. As the amount of the total sequence tags was 1 million, the new distinct tags were not detected any more (Figure S2 and Figure S6). Both results of the distribution of the clean tags in the two libraries showed that highly expressed genes had a broad distribution in the clean tags, while narrow in distinct clean tags (Table 1). By contrast, the fraction of genes with low expression in the clean tags is minimal, but was found high in distinct clean tags.

### Analysis of Tag Mapping

The mapping fraction of these distinct tag sequences to *Mus musculus* UniGene reference sequences could be seen in Figure S3. As polymorphism may exist in specimen, tolerances were defined to permit one mismatch in each alignment. Based on the criteria, 56.63% and 64.74% of distinct clean tags were mapped to the UniGene virtual tag database; 51.51% and 58.34% of the distinct clean tags were mapped unambiguously to the UniGene; 4.66% and 3.80% of the distinct clean tags did not match to the UniGene virtual tag database in the H37Rv and BCG-treated library, respectively (Table 1). The distribution of sample tag positions is shown in Figure S4. As essential qualities of sequencing tags, the sense and antisense strands of the transcripts can be distinguished using the Solexa sequencing. By comparison, the ratio of sense to antisense strand of the transcripts was approximately 1.99∶1 in the BCG-treated libraries, and in H37mRv-treated libraries was 1.75∶1 (Figure S5). In spite of the high number of antisense mapping events detected, the sense strand is of first importance on the part of the transcriptional regulation in the Mycobacterium tuberculosis-induced immune response.

### Gene Expression Variations between BCG-challenged and H37Rv-challenged Libraries

In order to search for gene expression variation between BCG-challenged and H37Rv-challenged libraries, the amount of clean tags for each gene was calculated, and the differently expressed genes between two samples were identified using a rigorous formula developed by Audic et al [22]. According to Figure 2, the number of significantly DEGs between two samples was 1,917, of which 1,135 genes and 782 genes were up-regulated and down-regulated, respectively. Specific information about DEGs can be seen in Table S2.

### Validation of DGE Data via qPCR

We randomly selected six DEGs: MyD88, Cd68, ULK1, TLR2, Arpc1b and TNF-α, to validate the correctness and confidence of DGE data. Data were presented as fold changes in gene expression normalized to GAPDH in Figure 3. Pearson’s correlation coefficient (r) showed that both the DGE and qPCR data were highly correlated, where the DEGs had a high consistency and the r values ranging from 0.681 (TLR2) to 0.995 (MyD88) between the two methods (Figure 2). The results show that the DGE results are dependable.

### KEGG and DEGs with Pathway Annotation

Among the 1,917 DEGs, 1,371 can be mapped into 210 pathways (Table S3). Based on the KEGG function classification, these mapped DEGs can be grouped into seven kinds of pathways: global pathway, metabolism, genetic information processing, environmental information processing, cellular processes, organismal systems, and human diseases. The pathways distribution showed in Figure 4. As can be seen, the human diseases pathways were showed as the highest amount of genes measured as 603 genes, while organismal systems pathways were 562. Meanwhile, genetic information processing pathways were 385, metabolism pathways were 383, cellular processes were 361. For the environmental information processing pathways and global pathways, the number was 224 and 212, respectively.

As the pathways are too much to study it, so we from our point of view selected 28 pathways based on the potential role, interaction with *Mycobacterium tuberculosis* and trying to make the selection cover all the stages of infection and immune response, then made further study on the selected pathways, where we grouped it into six categories based on KEGG, and illustrate its distribution as shown in Figure 5.

### Hierarchical Clustering and Tree View

The genes involved in the above 28 pathways and their expressions in three libraries (BCG treated, H37Rv treated and non-treated group as a control group) were analyzed using CLUSTER 3.0 available on the Internet and Tree View tool. The up regulated genes showed value over zero, while the down regulated genes showed value range from 0 to −2. The most up regulated genes belongs to the following pathways: cell cycle and apoptosis; MAPK signaling pathway, TGF-beta signaling pathway; ribosome; microbial metabolism in diverse environment; T cell receptor signaling pathway, antigen processing and presentation molecules. Meanwhile, the most down regulated genes belongs to the following pathways: phagosome, endocytosis and autophagy; calcium signaling pathway; metabolic pathways; Fc epsilon RI signaling pathway (as shown in Figure 6, Table S3, and Table S5).

### Gene Ontology (GO) Classification

We used the WEGO database to analysis our DEGs output, we made analysis for the whole 1917 DEGs, the analysis showed that 1767, 1730, 1722 genes maybe annotated in GO component, GO function, and GO process based on sequence homologies, respectively.

The DEGs from the selected 28 pathways were analyzed by the WEGO analysis. About 468, 466, and 468 genes could be annotated in GO component, GO function, and GO process based on sequence homologies, respectively. In the three main categories (cellular component, molecular function, and biological process) of the GO classification, “cell,” “cell part,” “binding,” “catalytic,” “metabolic process,” and “cellular process” were dominant (Figure 7, Figure S7 and Table S6).

### Pathogen Recognition Receptors

Toll-like receptors (TLR) and NOD-like receptors are main members in the pathogen recognition receptors family (PRRs). Compared with BCG, H37Rv can upregulate TLR1, TLR2, and TLR7 expression (Table 2). TLR2 is important for host to recognize lipoproteins. The expression of some nuclear factor just as Nfkbid, Nfkbiz, and Nfrkb were also upregulated in the H37Rv-treated library (Table S2).

### *Mycobacterium tuberculosis* Interaction with Macrophages

In order to survival in macrophage, Mycobacterium tuberculosis interacts with macrophage through several different receptors, including complement receptor (CR), FcγR, and TLR [29]. For the expression of complement receptor (CR), Cr1l was down-regulated, and for the expression of FcγR, Fcgr1 can be up-regulated in the H37Rv-treated library compared with the BCG-treated library (Table 2). MyD88 and Hmha1 involved in the TLR signaling pathway were downregulated in the H37Rv-treated library in comparison with the BCG-treated library (Table 2).

Pro-inflammatory cytokine signaling is crucial for an effective immune response against Mycobacterium tuberculosis [30]. The expression of IL-6, TNF-α, IL-15, tumor necrosis factor (Tnfaip3, Tnfaip6, and Tnfaip8), IFN-β1 as well as the cytokine receptor (TGF-βR1, Tnfrsf12a, Ripk1, Traf3, Traf6, and Traf7) were different between the H37Rv-treated and BCG-treated libraries (Table 2).

The comparative analysis between H37Rv-treated and BCG-treated library revealed significant expression for nine genes that involved in the delivery inhibition of vacuolar H+-ATPases to the phagosomes (Table S2). All these nine genes were downregulated in H37Rv-treated library compared with BCG-treated library. Among these nine down-regulated genes, seven involved in the ATPase H+ transporting (Atp5a1, Atp5d, Atp5h, Atp6ap, Atp6ap2, Atp6v0e2, and Atp5j2) (Table S2). The expression of actin-related protein expressing genes (Arpc1b and Arpc4) is also lower in the H37Rv library compared with that in the BCG library (Table S2). The disruption of actin can inhibit the phagosomal maturation, which is the best-characterized mechanism for Mycobacterium tuberculosis to manipulate the macrophage for survival and create a favorable environment for replication [31].

Compared with those in BCG-treated library, Cep110 and Cep55, which may contain early endosomal antigen 1 (EEA1), were down regulated in H37Rv-treated library (Table S2). In addition the expression of Rab5b is up regulated and Rab5c is down regulated in H37Rv challenged library (Table S2). Both Rab5 and EEA1 are necessary for phagosomal maturation to proceed [32], [33].

The expression of Med8 and Hmhal were down-regulated in H37Rv-treated library in comparison with BCG-treated library (Table S2), while the expression of Rab7 and Cd68 were up regulated and the expression of Scarb2 and Cd63 was down regulated in H37Rv-treated library in comparison with the BCG one (Table S2).

The expression of serine/threonine kinase, such as Ripk1, Pisd, Pxk, and Psat1, are different between the two libraries (Table S2). Among them, only Psat1 was highly downregulated, and all others were up regulated in the H37Rv-treatd library. Lysosomal hydrolases and serine/threonine kinase play an important role in regulating phagosomal maturation. Lysosomal hydrolases, such as cathepsins, glycosidases, sulfatase, lipase, and ceramidase were differently expressed between the two libraries. In 2007 Rohde K et al. demonstrated that PknG can regulate phagosomal maturation [34].

Autophagy regulation can eliminate Mycobacterium tuberculosis that resides within the phagosomes [16], [35]. Results showed that UlK1, Med8 and Hmhal were down regulated, while Atg7 and Atg12 were up regulated in H37Rv-treated group in compare with BCG one.

### Putative Novel Immune/stress Response Genes

The homology analysis showed that only 1828 transcripts had high sequence homology with the known sequences in the public database, while 89 transcripts didn’t show homology and couldn’t annotated clearly (Table S4), which maybe recommended it to be a putative novel immune related genes in RAW264.7 and involved in the *Mycobacterium tuberculosis* responses, which need further investigations and researches to make it more clear.

## Discussion

A timeless Solexa/Illumina’s DGE system was used to fully understand the whole transcriptional changes in the BCG-and H37Rv-challenged RAW264.7 with qPCR done to ensure the accuracy of the data. The transcriptome can explain all expressed RNA transcripts in a cell [9], [10]]. Furthermore, some cellular activities in organisms, such as disease and immune defense, can be well studied [9], [10].

There were 1,917 DEGs between H37Rv-challenged and BCG-challenged libraries (Figure 2, Table S2), around 1,135 genes up regulated while 782 genes down regulated, which suggested that these DEGs might be putative novel immune-relevant genes in RAW 264.7 involved in the response to *Mycobacterium tuberculosis* H37Rv and BCG challenges. Among the 1,917 DEGs, 1,371 mapped into 210 pathways (Table S3), grouped into seven kinds of pathways (Figure 4). Results suggested that after H37Rv or BCG infecting macrophages or possibly DCs, the global pathway, metabolism, genetic information processing, environmental information processing, cellular processes, and organismal systems all adopt some changes as shown above.

TLRs (TLR1-TLR13) are one of the signaling families of PRRs which can identify pathogen-associated molecular patterns (PAMPs) [36]. DGE analysis showed that the expression of TLR1, TLR2, and TLR7 were up-regulated at different levels in H37Rv-challenged library compared with that in BCG-challenged library (Figure 6E). TLR2 can interact with TLR1 or TLR6 to form heterodimers which can engage in the recognition of mycobacterial cell wall glycolipids, such as triacylated lipoproteins (TLR2/TLR1), or diacylated lipoproteins (TLR2/TLR6) [37]–[39]. This suggests that TLR-based immunity may participates in RAW264.7 defense against *Mycobacterium tuberculosis*.

In 2004 Gutierrez MG et al. demonstrated that autophagy was a defense mechanism inhibiting BCG and Mycobacterium tuberculosis survival in infected macrophages [16]. In 2007 Xu Y et al. demonstrated that the MyD88-independent gene was shown to be involved in lipopolysaccharide-induced autophagy [40]. Our DGE analysis showed the MyD88 transcript was down-regulated in the H37Rv-challenged library compared with that in BCG-challenged library. We make a forecast that as a consequence of their co-evolution, pathogens mimic host activities and regulation mechanisms to influence the autophagy by down-regulation of the MyD88 transcript (unpublished data).

The pro-inflammatory cytokine signaling is important for an accurate and effective immune response against *Mycobacterium tuberculosis*. DGE analysis demonstrated that the expression of IL-6, TNF-α and IL-15 were up-regulated in the H37Rv-treated library compared with those in BCG-treated library. If we want to improve present tuberculosis therapy, we may pay more attention to the function of these cytokine to effectively control *Mycobacterium tuberculosis* infection.

The well-known mechanism for Mycobacterium tuberculosis to evade the immunologic surveillance and the elimination by macrophage is to inhibit phagosome maturation. The delivery inhibition of vacuolar H+-ATPases to the phagosome may be the main reason for the delay of phagosome maturation [34]. The results showed that the expression of seven H+ transporting genes (Atp5a1, Atp5d, Atp5h, Atp6ap, Atp6ap2, Atp6v0e2, and Atp5j2) were all downregulated in H37Rv-challenged library. Thoroughly understanding the function of these important host genes may have important role in increasing the efficient of existing Mycobacterium tuberculosis vaccine (unpublished data).

During the interaction of phagosome and endosome, actin is essential for the scaffolding of endosomes [41]–[43]. DGE analysis demonstrated that the expression of actin-related protein expressing genes (Arpc1b and Arpc4) was also lower in H37Rv-challenged library. This suggests that Arpc1b and Arpc4 may be part of the reason for Mycobacterium tuberculosis-induced disruption of actin and the delay of phagosomal maturation [44], [45] .

EEA1 and Rho-GTPase Rab5 are also essential for phagosome maturation [46], [47]. The present results in H37Rv group showed that the expression of Cep110 and Cep55, which may be involved with EEA1, and Rab5c were down regulated, while Rab5b was up regulated in comparison with BCG one. We suspect that Cep110 and Cep55 may be part of the tethering factors, in concert with Rab proteins, specifically bridging membranes to be fused with Rab5 supporting early and Rab7 supporting late fusion events. Meanwhile we implicated that Rab5b and Rab5c have antagonistic roles on the trafficking of endosomal and reconciling the fusion of phagosome with other organelles (unpublished data).

Our results showed that there was down regulation for the UlK1 expression in H37Rv treated group in comparison with BCG ones (Table S2). Regulation of autophagy pathway analysis showed Ulk1 involved in the ATG 1 expression which has an important role in autophagy induction [48] (Table S3). This suggests that U1K1 may be part of the reason for Mycobacterium tuberculosis-induced the inhibition of autophagy induction.

The expression of Med8 and Hmhal were down-regulated in H37Rv-treated library in comparison with BCG-treated library (Table S2). We predicted according to regulation of autophagy pathway analysis (Table S3) that this may result PI3K down regulation which will inhibit the phagosomal maturation by influencing many fusion and fission events [49].

The Atg7 and Atg12 have important roles in docking and fusion, showed up regulation events in H37Rv-challenged library compared with that in BCG-challenged library (Table S2) [50]. This suggests that H37Rv do not make any further inhibion of the Vesicle expansion and completion in compared with BCG.

This study mainly used DGE to understand the interplay between *Mycobacterium tuberculosis* and RAW264.7. In a word, after H37Rv or BCG infecting macrophages or possibly DCs, the global pathway, metabolism, genetic information processing, environmental information processing, cellular processes, and organismal systems all adopt some changes as shown above. As *Mycobacterium tuberculosis* has evolved an array of mechanisms to allow survival within the macrophage, we just want to find some important host protein which was affected by *Mycobacterium tuberculosis* to provide evidence for the further improvement of the efficacy of existing *Mycobacterium tuberculosis* therapy and vaccine.

## Supporting Information

Figure S1 Distribution of total clean tags and distinct clean tags in experimental and control library. The numbers in square brackets indicate the range of copy numbers of each tag category. The data in parentheses indicate the percentage of corresponding tags among the total clean tags and distinct clean tags. The results confirmed that the quality of the data were up to specification suggesting that DGE data are significant.
(TIF)

Figure S2 Saturation of DGE libraries. The saturation analysis of capacity of libraries showed that new emerging distinct tags were gradually decreased with increasing total sequence tags when the number of sequencing tags was high enough. (A) H37Rv-treated library; (B) BCG-treated library; (C) control library. In saturation analysis of the capacity of libraries, with more and more sequence tags, the amount of new distinct tags was decreasing, in the condition that the amount of total sequence tags was big enough. As the amount of the total sequence tags was 1 million, the new distinct tags were not detected any more. Results suggested that the saturation of DGE libraries fulfill the need of DGE’s requirement.
(TIF)

Figure S3 Mapping of total and distinct clean tags. The mapping fraction of these distinct tag sequences to *Mus musculus* UniGene reference sequences could be seen in Figure S3.
(TIF)

Figure S4 The positions of tags in the gene. A) the H37Rv-infected library; (B) the BCG-infected library; (C) the control library. Ideally, the tag is the 3 most one. But for alternative splicing or incomplete enzyme digestion, the tag may be the 2^nd^ or 3^rd^ from the 3 most.
(TIF)

Figure S5 Sense vs antisense transcripts expression in the experimental and control library. (A) H37Rv-infected library; (B) BCG-infected library; (C) control library. As essential qualities of sequencing tags, the sense and antisense strands of the transcripts can be distinguished using the Solexa sequencing. By comparison, the ratio of sense to antisense strand of the transcripts was approximately 1.99∶1 in the BCG-treated libraries, and in H37Rv-treated libraries was 1.75∶1 as shown in Figure S5. Results suggested that in spite of the high number of antisense mapping events detected, the sense strand is of first importance on the part of the transcriptional regulation in the *Mycobacterium tuberculosis*-induced immune response.
(TIF)

Figure S6 Effect of library size on the number of genes identified. (A) H37Rv-infected library; (B) BCG-infected library; (C) contral library. The increasing tendency in the rate of increase in all genes identified and the genes identified by unambigous tags declined with increasing library size. The rate of increase of all genes identified and genes identified by unambigous clean tags declined drastically as the size of the library increased. When the library size reached one million, we could identify 35% and 30% all genes and genes identified by unambigous clean tags, in (A) H37Rv-infected library and (B) BCG-infected library, respectively. Simultaneously, we could identify 48% and 30% all genes and genes identified by unambigous clean tags, in (C) control library. At this time, library capacity approached saturation. Results suggested that the saturation of DGE libraries fulfill the need of DGE’s requirement.
(TIF)

Figure S7 Signaling pathways of the DEGs. The pathway analysis was mainly based on the KEGG database. During gene ontology analysis, the gene sets with *P*<0.1 should be deemed significantly enriched. An FDR of <0.1 must be considered for the KEGG enrichment analysis. The vertical axis is the pathway category, and the horizontal axis is the log10 (p value) of these pathways. We from our point of view selected 28 pathways, which may have some potential function for in-depth studies on *Mycobacterium tuberculosis*. Results suggested that in these 28 selected pathways the DGEs mainly distributed in global pathways, cellular processes pathways and organismal Systems pathways.
(TIF)

Table S1 The qPCR primer sequences.
(XLS)

Table S2 The whole information about DEGs in the H37Rv-infected library and BCG-infected library. Each column stands for: the number in “gene” represented Gene ID; Raw Intensity, Raw clean tag number; TPM (Transcripts Per Million clean tags) is a standardized indicator, pointing out number of transcript copies in every 1 million clean tags. This is Sum of the normalized clean tag number in Table S2. Symbol, Gene symbol; Description, Brief gene description; GO Component, Ontology Information of Cellular Components of Gene-corresponding GO terms; GO Function, Ontology Information of Molecular Functions of Gene-corresponding GO terms; GO Process, Ontology Information of Biological Processes of Gene-corresponding GO terms; transcriptID, Gene Names in Transcripts Sequences.
(XLS)

Table S3 Pathway annotation of DEGs in H37Rv-infected library and BCG-infected library based on KEGG. Each column stands for: sample1 and sample2 represented BCG-infected library and H37Rv-infected library, separately; Pvalue, P-value in Hypergeometric Test; Qvalue, Qvalue (Pathways with Qvalue≤0.05 are significantly enriched in DEGs); the number in “gene”, Gene ID; KOs, Pathway ID.
(XLS)

Table S4 Potential pathway annotation of putative novel immune-relevant DGEs in RAW 264.7 involved in the response to the *Mycobacterium tuberculosis* challenge based on KEGG. What each column stands for just as shown in Table S4. These 28 pathways were selected from our point of view which may have some potential function for in-depth studies on *Mycobacterium tuberculosis*. Results suggested that in these 28 selected pathways the DGEs mainly distributed in global pathways, cellular processes pathways and organismal Systems pathways. In addition, more attention may be given to these *Mycobacterium tuberculosis*-affected immune-related genes in RAW264.7.
(XLS)

Table S5 KEGG categorization of potential pathway based on KEGG. Results showed the number of DEGs in every pathway which belongs to the KEGG categorization among these 28 pathways selected from our point of view which may have some potential function for the in-depth studies on *Mycobacterium tuberculosis*.
(XLS)

Table S6 Complete information on the GO annotation on DEGs belonging to the potential pathway. What each column stands for just as shown in Table S2. The DEGs from the selected 28 pathways were analyzed by the WEGO analysis. About 468, 466, and 468 genes could be annotated in GO component, GO function, and GO process based on sequence homologies, respectively. In the three main categories (cellular component, molecular function, and biological process) of the GO classification, “cell,” “cell part,” “binding,” “catalytic,” “metabolic process,” and “cellular process” were dominant.
(XLS)

## References

[1] Coler RN, Dillon DC, Skeiky YA, Kahn M, Orme IM, et al (2009) Identification of Mycobacterium tuberculosis vaccine candidates using human CD4+ T-cells expression cloning. Vaccine 27: 223–233. 19000730 10.1016/j.vaccine.2008.10.056 PMC2673800

[2] Dye C, Williams BG (2010) The population dynamics and control of tuberculosis. Science 328: 856–861. 20466923 10.1126/science.1185449

[3] Lee J, Hartman M, Kornfeld H (2009) Macrophage apoptosis in tuberculosis. Yonsei Med J 50: 1–11. 19259342 10.3349/ymj.2009.50.1.1 PMC2649858

[4] Warner DF, Mizrahi V (2006) Tuberculosis chemotherapy: the influence of bacillary stress and damage response pathways on drug efficacy. Clin Microbiol Rev 19: 558–570. 16847086 10.1128/CMR.00060-05 PMC1539104

[5] Oliveira HB, Mateus SH (2011) [Characterization of multidrug-resistant tuberculosis during pregnancy in Campinas, State of Sao Paulo, Brazil, from 1995 to 2007]. Rev Soc Bras Med Trop 44: 627–630. 22031080

[6] Ward SK, Abomoelak B, Marcus SA, Talaat AM (2010) Transcriptional profiling of mycobacterium tuberculosis during infection: lessons learned. Front Microbiol 1: 121. 21738523 10.3389/fmicb.2010.00121 PMC3125582

[7] Sanarico N, Colone A, Grassi M, Speranza V, Giovannini D, et al (2011) Different transcriptional profiles of human monocyte-derived dendritic cells infected with distinct strains of Mycobacterium tuberculosis and Mycobacterium bovis bacillus Calmette-Guerin. Clin Dev Immunol 2011: 741051. 21436989 10.1155/2011/741051 PMC3062957

[8] Ansari MA, Zubair S, Mahmood A, Gupta P, Khan AA, et al (2011) RD antigen based nanovaccine imparts long term protection by inducing memory response against experimental murine tuberculosis. PLoS One 6: e22889. 21853054 10.1371/journal.pone.0022889 PMC3154911

[9] Hegedus Z, Zakrzewska A, Agoston VC, Ordas A, Racz P, et al (2009) Deep sequencing of the zebrafish transcriptome response to mycobacterium infection. Mol Immunol 46: 2918–2930. 19631987 10.1016/j.molimm.2009.07.002

[10] Stockhammer OW, Zakrzewska A, Hegedus Z, Spaink HP, Meijer AH (2009) Transcriptome profiling and functional analyses of the zebrafish embryonic innate immune response to Salmonella infection. J Immunol 182: 5641–5653. 19380811 10.4049/jimmunol.0900082

[11] Wang Z, Gerstein M, Snyder M (2009) RNA-Seq: a revolutionary tool for transcriptomics. Nat Rev Genet 10: 57–63. 19015660 10.1038/nrg2484 PMC2949280

[12] Anisimov SV (2008) Serial Analysis of Gene Expression (SAGE): 13 years of application in research. Curr Pharm Biotechnol 9: 338–350. 18855686 10.2174/138920108785915148

[13] Xiang LX, He D, Dong WR, Zhang YW, Shao JZ (2010) Deep sequencing-based transcriptome profiling analysis of bacteria-challenged Lateolabrax japonicus reveals insight into the immune-relevant genes in marine fish. BMC Genomics 11: 472. 20707909 10.1186/1471-2164-11-472 PMC3091668

[14] Wang B, Guo G, Wang C, Lin Y, Wang X, et al (2010) Survey of the transcriptome of Aspergillus oryzae via massively parallel mRNA sequencing. Nucleic Acids Res 38: 5075–5087. 20392818 10.1093/nar/gkq256 PMC2926611

[15] Chin MP, Schauer DB, Deen WM (2010) Nitric oxide, oxygen, and superoxide formation and consumption in macrophages and colonic epithelial cells. Chem Res Toxicol 23: 778–787. 20201482 10.1021/tx900415k PMC2879621

[16] Gutierrez MG, Master SS, Singh SB, Taylor GA, Colombo MI, et al (2004) Autophagy is a defense mechanism inhibiting BCG and Mycobacterium tuberculosis survival in infected macrophages. Cell 119: 753–766. 15607973 10.1016/j.cell.2004.11.038

[17] Christophe T, Jackson M, Jeon HK, Fenistein D, Contreras-Dominguez M, et al (2009) High content screening identifies decaprenyl-phosphoribose 2′ epimerase as a target for intracellular antimycobacterial inhibitors. PLoS Pathog 5: e1000645. 19876393 10.1371/journal.ppat.1000645 PMC2763345

[18] Kozak RA, Alexander DC, Liao R, Sherman DR, Behr MA (2011) Region of difference 2 contributes to virulence of Mycobacterium tuberculosis. Infect Immun 79: 59–66. 20974821 10.1128/IAI.00824-10 PMC3019914

[19] Deb C, Lee CM, Dubey VS, Daniel J, Abomoelak B, et al (2009) A novel in vitro multiple-stress dormancy model for Mycobacterium tuberculosis generates a lipid-loaded, drug-tolerant, dormant pathogen. PLoS One 4: e6077. 19562030 10.1371/journal.pone.0006077 PMC2698117

[20] Xiao S, Jia J, Mo D, Wang Q, Qin L, et al (2010) Understanding PRRSV infection in porcine lung based on genome-wide transcriptome response identified by deep sequencing. PLoS One 5: e11377. 20614006 10.1371/journal.pone.0011377 PMC2894071

[21] Xue J, Bao YY, Li BL, Cheng YB, Peng ZY, et al (2010) Transcriptome analysis of the brown planthopper Nilaparvata lugens. PLoS One 5: e14233. 21151909 10.1371/journal.pone.0014233 PMC2997790

[22] Audic S, Claverie JM (1997) The significance of digital gene expression profiles. Genome Res 7: 986–995. 9331369 10.1101/gr.7.10.986

[23] Kissner TL, Ruthel G, Alam S, Ulrich RG, Fernandez S, et al.. (2011) Activation of MyD88 Signaling upon Staphylococcal Enterotoxin Binding to MHC Class II Molecules. PLoS One 6. 10.1371/journal.pone.0015985 PMC3024394 21283748

[24] Weigelt K, Ernst W, Walczak Y, Ebert S, Loenhardt T, et al (2007) Dap12 expression in activated microglia from retinoschisin-deficient retina and its PU.1-dependent promoter regulation. Journal of Leukocyte Biology 82: 1564–1574. 17827340 10.1189/jlb.0707447

[25] Hara T, Takamura A, Kishi C, Iemura SI, Natsume T, et al (2008) FIP200, a ULK-interacting protein, is required for autophagosome formation in mammalian cells. Journal of Cell Biology 181: 497–510. 18443221 10.1083/jcb.200712064 PMC2364687

[26] Friberg IM, Lowe A, Ralli C, Bradley JE, Jackson JA (2011) Temporal Anomalies in Immunological Gene Expression in a Time Series of Wild Mice: Signature of an Epidemic? PLoS One 6. 10.1371/journal.pone.0020070 PMC3100328 21629775

[27] Noel S, Sharma S, Shanker R, Rath SK (2007) Primaquine-induced differential gene expression analysis in mice liver using DNA microarrays. Toxicology 239: 96–107. 17686563 10.1016/j.tox.2007.06.098

[28] Kanehisa M, Araki M, Goto S, Hattori M, Hirakawa M, et al (2008) KEGG for linking genomes to life and the environment. Nucleic Acids Res 36: D480–484. 18077471 10.1093/nar/gkm882 PMC2238879

[29] Greenberg S, Grinstein S (2002) Phagocytosis and innate immunity. Curr Opin Immunol 14: 136–145. 11790544 10.1016/s0952-7915(01)00309-0

[30] Bulut Y, Michelsen KS, Hayrapetian L, Naiki Y, Spallek R, et al (2005) Mycobacterium tuberculosis heat shock proteins use diverse toll-like receptor pathways to activate pro-inflammatory signals. Journal of Biological Chemistry 280: 20961–20967. 15809303 10.1074/jbc.M411379200

[31] Stewart GR, Patel J, Robertson BD, Rae A, Young DB (2005) Mycobacterial mutants with defective control of phagosomal acidification. Plos Pathogens 1: 269–278. 16322769 10.1371/journal.ppat.0010033 PMC1291353

[32] Deretic V, Singh S, Master S, Harris J, Roberts E, et al (2006) Mycobacterium tuberculosis inhibition of phagolysosome biogenesis and autophagy as a host defence mechanism. Cellular Microbiology 8: 719–727. 16611222 10.1111/j.1462-5822.2006.00705.x

[33] Weber SS, Ragaz C, Hilbi H (2009) Pathogen trafficking pathways and host phosphoinositide metabolism. Molecular Microbiology 71: 1341–1352. 19208094 10.1111/j.1365-2958.2009.06608.x

[34] Rohde K, Yates RM, Purdy GE, Russell DG (2007) Mycobacterium tuberculosis and the environment within the phagosome. Immunol Rev 219: 37–54. 17850480 10.1111/j.1600-065X.2007.00547.x

[35] Deretic V, Delgado M, Vergne I, Master S, De Haro S, et al (2009) Autophagy in Immunity Against Mycobacterium tuberculosis: a Model System to Dissect Immunological Roles of Autophagy. Autophagy in Infection and Immunity 335: 169–188. 10.1007/978-3-642-00302-8_8 PMC2788935 19802565

[36] Korbel DS, Schneider BE, Schaible UE (2008) Innate immunity in tuberculosis: myths and truth. Microbes Infect 10: 995–1004. 18762264 10.1016/j.micinf.2008.07.039

[37] Means TK, Jones BW, Schromm AB, Shurtleff BA, Smith JA, et al (2001) Differential effects of a Toll-like receptor antagonist on Mycobacterium tuberculosis-induced macrophage responses. J Immunol 166: 4074–4082. 11238656 10.4049/jimmunol.166.6.4074

[38] Jones BW, Means TK, Heldwein KA, Keen MA, Hill PJ, et al (2001) Different Toll-like receptor agonists induce distinct macrophage responses. J Leukoc Biol 69: 1036–1044. 11404392

[39] Thoma-Uszynski S, Stenger S, Takeuchi O, Ochoa MT, Engele M, et al (2001) Induction of direct antimicrobial activity through mammalian toll-like receptors. Science 291: 1544–1547. 11222859 10.1126/science.291.5508.1544

[40] Xu Y, Jagannath C, Liu XD, Sharafkhaneh A, Kolodziejska KE, et al (2007) Toll-like receptor 4 is a sensor for autophagy associated with innate immunity. Immunity 27: 135–144. 17658277 10.1016/j.immuni.2007.05.022 PMC2680670

[41] Hestvik AL, Hmama Z, Av-Gay Y (2005) Mycobacterial manipulation of the host cell. FEMS Microbiol Rev 29: 1041–1050. 16040149 10.1016/j.femsre.2005.04.013

[42] Guerin I, de Chastellier C (2000) Pathogenic mycobacteria disrupt the macrophage actin filament network. Infect Immun 68: 2655–2662. 10768957 10.1128/iai.68.5.2655-2662.2000 PMC97472

[43] Guerin I, de Chastellier C (2000) Disruption of the actin filament network affects delivery of endocytic contents marker to phagosomes with early endosome characteristics: the case of phagosomes with pathogenic mycobacteria. Eur J Cell Biol 79: 735–749. 11089922 10.1078/0171-9335-00092

[44] Esposito MS, Esposito RE, Arnaud M, Halvorson HO (1969) Acetate utilization and macromolecular synthesis during sporulation of yeast. J Bacteriol 100: 180–186. 5344095 10.1128/jb.100.1.180-186.1969 PMC315375

[45] Guerin I, de Chastellier C (2000) Disruption of the actin filament network affects delivery of endocytic contents marker to phagosomes with early endosome characteristics: The case of phagosomes with pathogenic mycobacteria. European Journal of Cell Biology 79: 735–749. 11089922 10.1078/0171-9335-00092

[46] Vergne I, Chua J, Singh SB, Deretic V (2004) Cell biology of mycobacterium tuberculosis phagosome. Annu Rev Cell Dev Biol 20: 367–394. 15473845 10.1146/annurev.cellbio.20.010403.114015

[47] Perskvist N, Roberg K, Kulyte A, Stendahl O (2002) Rab5a GTPase regulates fusion between pathogen-containing phagosomes and cytoplasmic organelles in human neutrophils. J Cell Sci 115: 1321–1330. 11884531 10.1242/jcs.115.6.1321

[48] Chang YY, Neufeld TP (2009) An Atg1/Atg13 complex with multiple roles in TOR-mediated autophagy regulation. Mol Biol Cell 20: 2004–2014. 19225150 10.1091/mbc.E08-12-1250 PMC2663935

[49] Harrison RE, Bucci C, Vieira OV, Schroer TA, Grinstein S (2003) Phagosomes fuse with late endosomes and/or lysosomes by extension of membrane protrusions along microtubules: role of Rab7 and RILP. Mol Cell Biol 23: 6494–6506. 12944476 10.1128/MCB.23.18.6494-6506.2003 PMC193691

[50] Fader CM, Colombo MI (2009) Autophagy and multivesicular bodies: two closely related partners. Cell Death Differ 16: 70–78. 19008921 10.1038/cdd.2008.168

