# Supplementary figures and images for: Different Transcriptional Profiles of RAW264.7 Infected with Mycobacterium tuberculosis H37Rv and BCG Identified via Deep Sequencing
Source: PLoS One. 2012 Dec 19;7(12):e51988. doi: 10.1371/journal.pone.0051988 (PMC3526534; doi:10.1371/journal.pone.0051988)

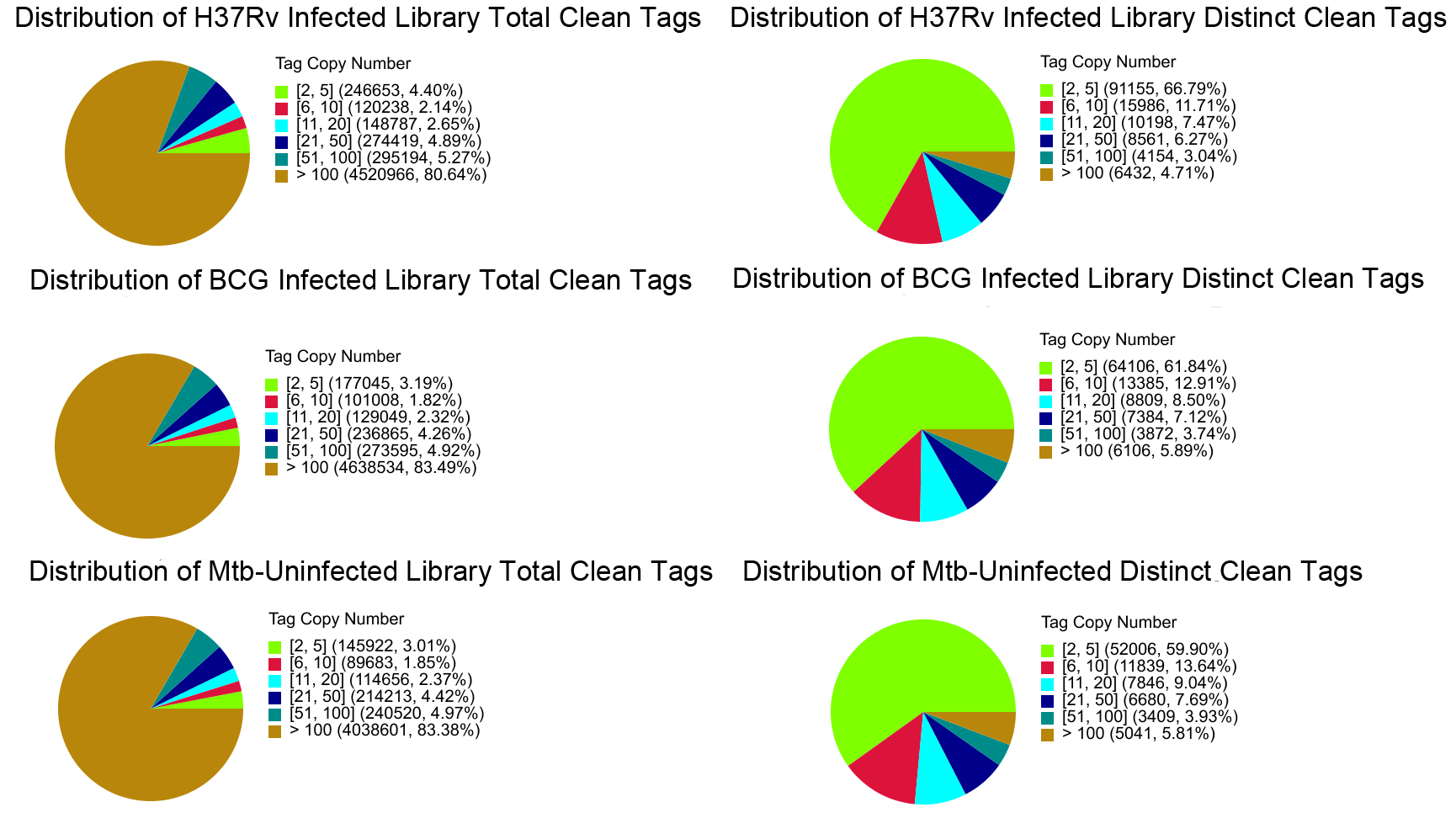

Supplement: Figure S1 — Distribution of total clean tags and distinct clean tags in experimental and control library. The numbers in square brackets indicate the range of copy numbers of each tag category. The data in parentheses indicate the percentage of corresponding tags among the total clean tags and distinct clean tags. The results confirmed that the quality of the data were up to specification suggesting that DGE data are significant. (TIF) [file pone.0051988.s001.tif]

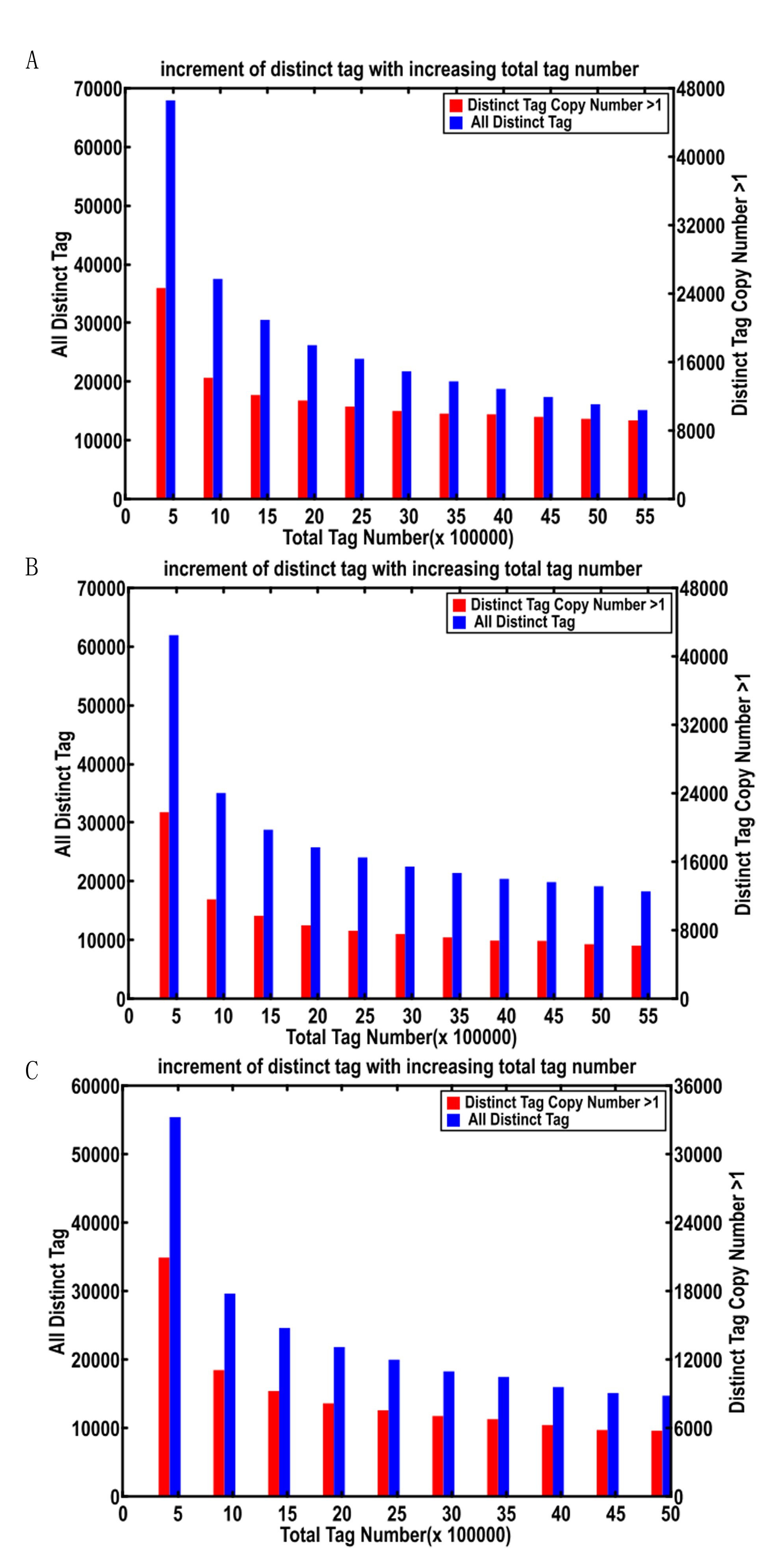

Supplement: Figure S2 — Saturation of DGE libraries. The saturation analysis of capacity of libraries showed that new emerging distinct tags were gradually decreased with increasing total sequence tags when the number of sequencing tags was high enough. (A) H37Rv-treated library; (B) BCG-treated library; (C) control library. In saturation analysis of the capacity of libraries, with more and more sequence tags, the amount of new distinct tags was decreasing, in the condition that the amount of total sequence tags was big enough. As the amount of the total sequence tags was 1 million, the new distinct tags were not detected any more. Results suggested that the saturation of DGE libraries fulfill the need of DGE’s requirement. (TIF) [file pone.0051988.s002.tif]

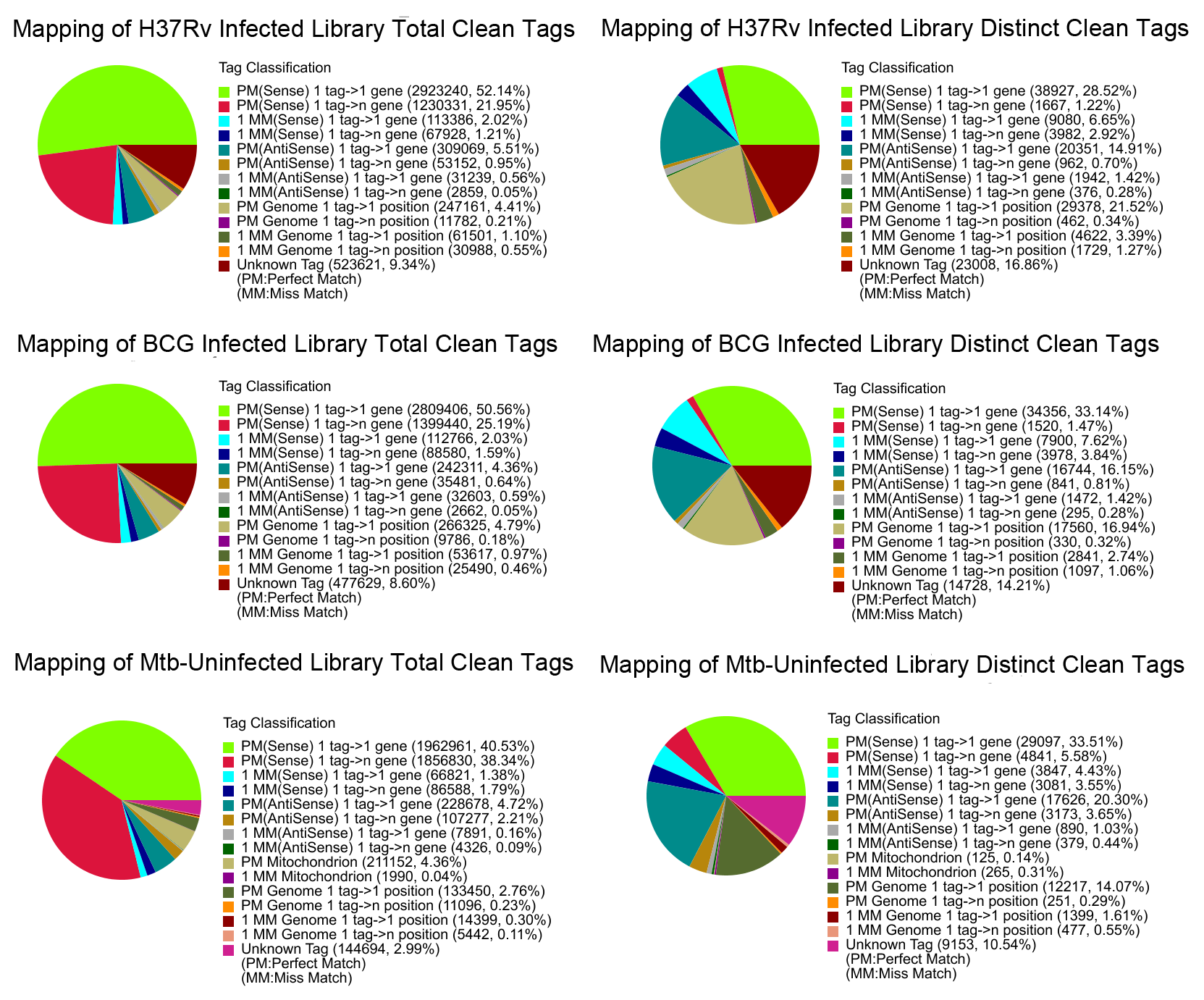

Supplement: Figure S3 — Mapping of total and distinct clean tags. The mapping fraction of these distinct tag sequences to Mus musculus UniGene reference sequences could be seen in Figure S3. (TIF) [file pone.0051988.s003.tif]

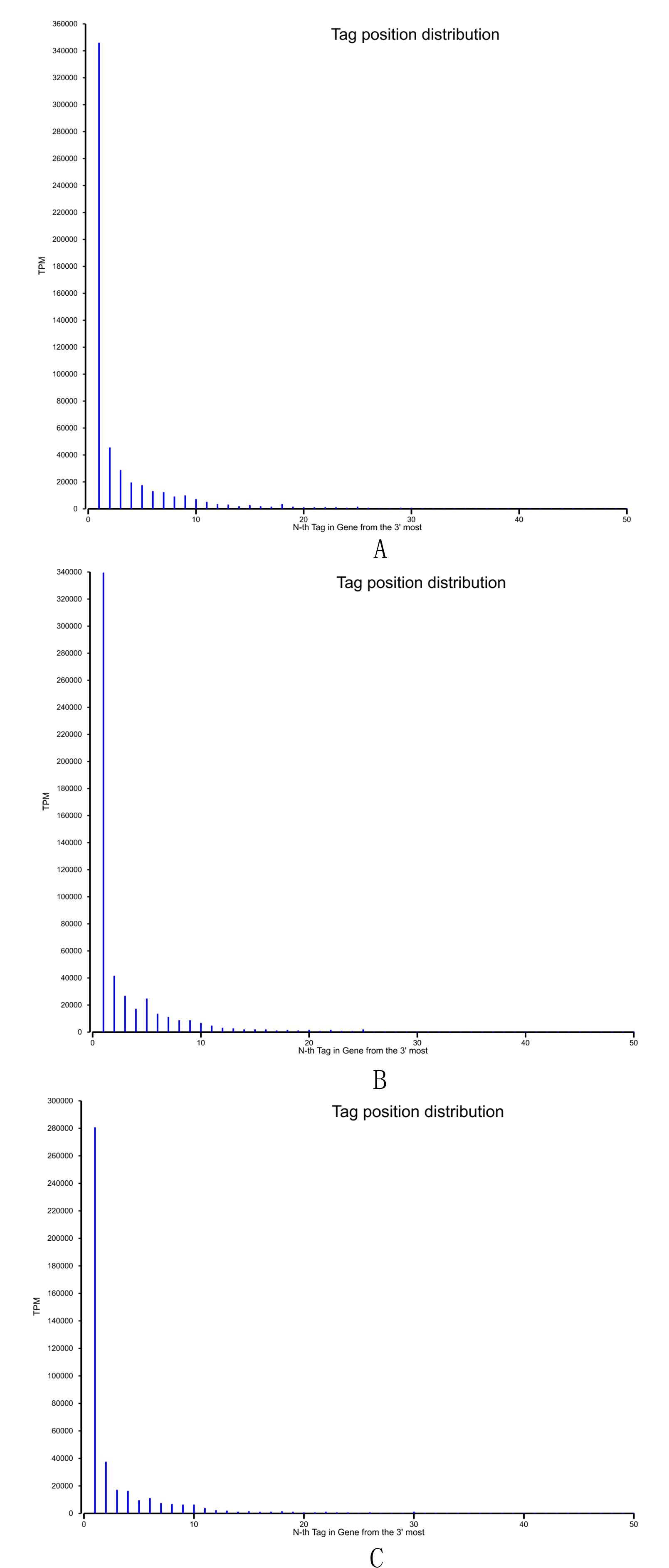

Supplement: Figure S4 — The positions of tags in the gene. A) the H37Rv-infected library; (B) the BCG-infected library; (C) the control library. Ideally, the tag is the 3 most one. But for alternative splicing or incomplete enzyme digestion, the tag may be the 2nd or 3rd from the 3 most. (TIF) [file pone.0051988.s004.tif]

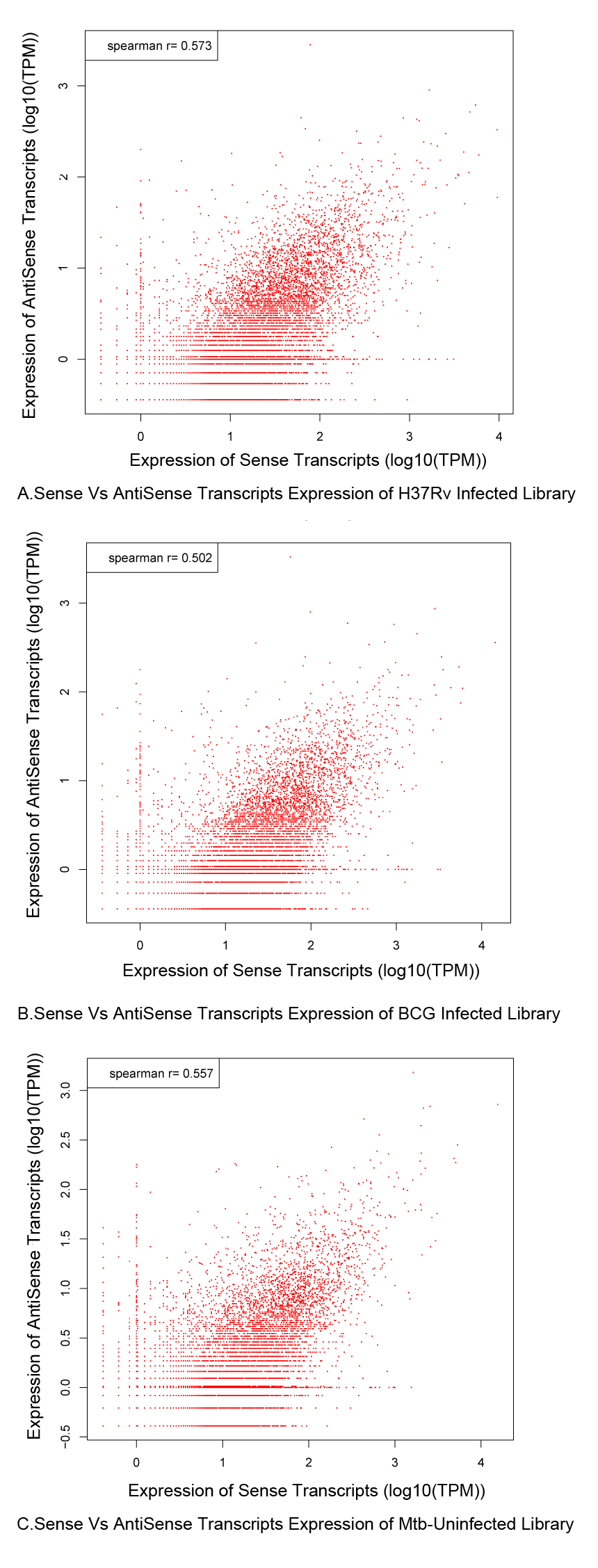

Supplement: Figure S5 — Sense vs antisense transcripts expression in the experimental and control library. (A) H37Rv-infected library; (B) BCG-infected library; (C) control library. As essential qualities of sequencing tags, the sense and antisense strands of the transcripts can be distinguished using the Solexa sequencing. By comparison, the ratio of sense to antisense strand of the transcripts was approximately 1.99∶1 in the BCG-treated libraries, and in H37Rv-treated libraries was 1.75∶1 as shown in Figure S5. Results suggested that in spite of the high number of antisense mapping events detected, the sense strand is of first importance on the part of the transcriptional regulation in the Mycobacterium tuberculosis-induced immune response. (TIF) [file pone.0051988.s005.tif]

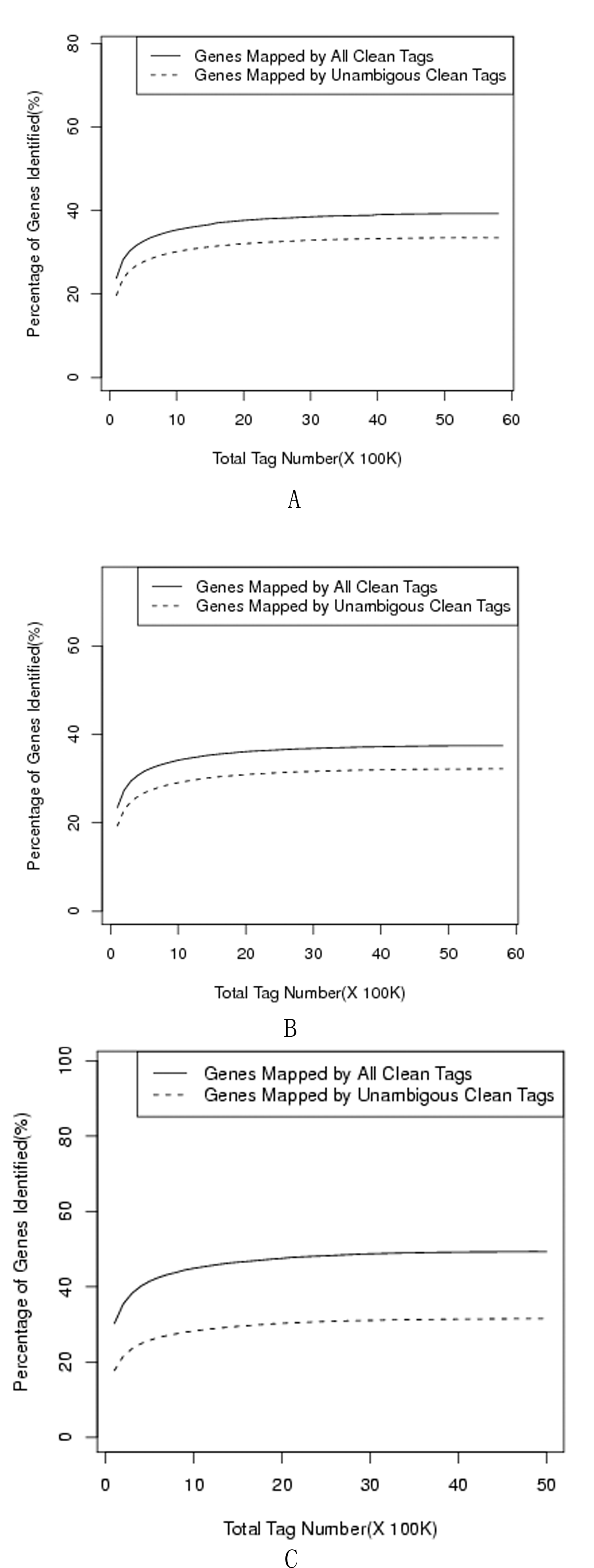

Supplement: Figure S6 — Effect of library size on the number of genes identified. (A) H37Rv-infected library; (B) BCG-infected library; (C) contral library. The increasing tendency in the rate of increase in all genes identified and the genes identified by unambigous tags declined with increasing library size. The rate of increase of all genes identified and genes identified by unambigous clean tags declined drastically as the size of the library increased. When the library size reached one million, we could identify 35% and 30% all genes and genes identified by unambigous clean tags, in (A) H37Rv-infected library and (B) BCG-infected library, respectively. Simultaneously, we could identify 48% and 30% all genes and genes identified by unambigous clean tags, in (C) control library. At this time, library capacity approached saturation. Results suggested that the saturation of DGE libraries fulfill the need of DGE’s requirement. (TIF) [file pone.0051988.s006.tif]
